# *Porphyromonas gingivalis* GroEL Accelerates Abdominal Aortic Aneurysm Formation by Induction of M1 Polarization in Macrophages

**DOI:** 10.3390/ijms26167781

**Published:** 2025-08-12

**Authors:** Yi-Wen Lin, Yi-Ting Tsai, Ming-Jen Cheng, Chun-Ming Shih, Chun-Yao Huang, Chien-Sung Tsai, Shih-Ying Sung, Ze-Hao Lai, Chen-Wei Liu, Feng-Yen Lin

**Affiliations:** 1 Institute of Oral Biology, National Yang Ming Chiao Tung University (Taipei Campus), Taipei 112, Taiwan; ywlin@nycu.edu.tw (Y.-W.L.); ljh85919@gmail.com (Z.-H.L.); 2 Taipei Heart Institute, Taipei Medical University, Taipei 110, Taiwan; cmshih53@tmu.edu.tw (C.-M.S.); cyhuang@tmu.edu.tw (C.-Y.H.); sung1500@mail.ndmctsgh.edu.tw (C.-S.T.); 3 Division of Cardiovascular Surgery, Tri-Service General Hospital, National Defense Medical University, Taipei 115, Taiwan; cvsallen@mail.ndmctsgh.edu.tw (Y.-T.T.); molecule1983@gmail.com (S.-Y.S.); 4 Department of Life Science, Fu Jen Catholic University, New Taipei City 242, Taiwan; 155410@mail.fju.edu.tw; 5 Division of Cardiology and Cardiovascular Research Center, Taipei Medical University Hospital, Taipei 110, Taiwan; 6 Division of Cardiology, Department of Internal Medicine, School of Medicine, College of Medicine, Taipei Medical University, Taipei 110, Taiwan; 7 Department of Biomedical Sciences and Engineering, National Central University, Taoyuan 320, Taiwan; 8 Department and Graduate Institute of Pharmacology, National Defense Medical University, Taipei 115, Taiwan; 9 Department of Basic Medical Science, College of Medicine, University of Arizona, Phoenix, AZ 85721, USA; cwliu0617@arizona.edu

**Keywords:** abdominal aortic aneurysm, macrophages, *P. gingivalis*, GroEL, thrombomodulin, IRF5

## Abstract

Abdominal aortic aneurysm (AAA) is a life-threatening vascular disease characterized by chronic inflammation, extracellular matrix degradation, and smooth muscle cell apoptosis. *Porphyromonas gingivalis* (*P. gingivalis*), a key periodontal pathogen, has been implicated in the progression of cardiovascular diseases, including AAA, but the underlying mechanisms remain unclear. In this study, we investigated the role of GroEL, a bacterial heat shock protein 60 homolog derived from *P. gingivalis*, in AAA development. We employed a CaCl_2_-induced AAA mouse model to evaluate the in vivo effects of GroEL. Mice received periaortic CaCl_2_ application followed by intravenous injections of recombinant GroEL. Histological analyses were performed to assess aneurysmal dilation, elastin degradation, and inflammatory cell infiltration. Flow cytometry and immunohistochemistry were used to determine macrophage phenotypes, while cytokine profiles were quantified via ELISA. In vitro, THP-1 monocytes were treated with GroEL to evaluate its impact on macrophage polarization and cytokine expression. Our results showed that GroEL administration significantly enhanced aortic diameter expansion and elastin breakdown, accompanied by increased infiltration of M1-like macrophages and elevated levels of pro-inflammatory cytokines such as TNF-α and IL-6. In vitro findings confirmed that GroEL promotes M1 polarization and inhibits M2 marker expression in THP-1-derived macrophages. These findings suggest that *P. gingivalis*-derived GroEL plays a pathogenic role in AAA by modulating macrophage polarization toward a pro-inflammatory phenotype. Targeting microbial components such as GroEL may offer new therapeutic strategies for AAA management.

## 1. Introduction

Oral hygiene and periodontitis severity are related to the occurrence of aortic aneurysms [1]. In fact, clinical research conducted by Suzuki et al. showed that periodontal pathogen infection has a greater influence on the course of aneurysms than other cardiovascular diseases [2,3]. Additionally, an abdominal aortic aneurysm (AAA) refers to a pathological dilation of the abdominal aorta that arises from structural weakening of the vessel wall. Without timely intervention, the aneurysm may progressively enlarge and eventually rupture, resulting in a life-threatening hemorrhage and a high risk of mortality [4]. Previously, Ishikawa et al. analyzed specimens of patients undergoing AAA surgery between 2001 and 2003 and found that a high proportion of samples contained periodontal pathogens [5]. Their study analyzed these periodontal pathogens and found that patients with *P. gingivalis*-induced periodontitis had the highest probability of *P. gingivalis* accumulation in their aortic specimens. Substantiating this, polymerase chain reaction analysis of human AAA specimens detected the presence of *P. gingivalis* DNA, primarily located in the intraluminal thrombus and aneurysmal wall [4,5]. However, Kręgielczak et al. argued that the detection of *P. gingivalis* in vascular tissues might be incidental rather than indicative of active infection or pathogenic involvement [6]. This is corroborated by the fact that anaerobic bacteria can be delivered by blood circulation and reach the intraluminal thrombus due to their direct link with the lumen of the vessel and the wall surface of the AAA. Therefore, periodontal pathogens and AAA are considered to be closely related [5,7,8].

The process of microbial intrusion on the endothelial intraluminal thrombus appears to involve the fimbriae of *P. gingivalis* [9,10]; this clarifies the detection of *P. gingivalis* DNA in AAA samples. In this process, the fimbriae from *P. gingivalis* anchorage induce the expression of interleukin (IL)-8 and chemotaxis of monocytes [11,12], as well as increase the number of neutrophils located in the intraluminal thrombus. *P. gingivalis* promotes the formation of neutrophil extracellular traps (NETs) at the interface between the intraluminal thrombus and the circulating blood, creating a dense network that contributes to thrombus stabilization and expansion. This NET-mediated environment facilitates sustained inflammation and thrombus growth, thereby accelerating the progression of AAA [13].

AAA progression also involves the destruction of elastic fibers due to high protease activity. Previous studies have found that the aortic walls of animals infected with *P. gingivalis* show greater matrix metalloproteinase (MMP)-9 activity, suggesting greater proteolytic activity in the luminal layer of the intraluminal thrombus [14,15]. Moreover, *P. gingivalis* infection entails a substantial rise in myeloperoxidase-DNA complexes in both plasma and AAA samples [13], causing oxidative stress. Oxidative stress participates in AAA pathogenesis by contributing to the dysregulation of MMPs and apoptosis of smooth muscle cells (SMCs) [16,17,18]. Therefore, although a limited number of previous studies have evaluated the relationship between AAA and periodontitis, there is evidence that periodontal microorganisms in the bloodstream or infiltrating the vascular lesion are associated with AAA progression.

GroEL is a heat shock protein 60 (HSP60) chaperonin produced by *P. gingivalis*, a bacterium implicated in periodontal disease. Beyond its role in protein folding, GroEL has been identified as a virulence factor contributing to various pathogenic processes. Since 2014, our laboratory has studied the effects of *P. gingivalis* on tumor angiogenesis, atherogenesis, and periodontal disease. We found that its heat-shock protein, GroEL, enhances endothelial nitric oxide synthase (eNOS) and p38 MAPK activity, promoting angiogenesis and tumor growth [19]. GroEL also induces LOX-1, ICAM-1, and VCAM-1 in vascular endothelial cells, increasing cholesterol uptake and monocyte adhesion, contributing to atherosclerosis [20]. Additionally, GroEL activates monocytes, enhances inflammatory cytokine production, and stimulates osteoclasts [21]. Our research suggests that *P. gingivalis* GroEL triggers systemic inflammatory diseases, including atherosclerosis and AAA. Even after treatment, bacterial components like LPS and GroEL persist, inducing chronic inflammation. Understanding these mechanisms may help combat *P. gingivalis*-associated diseases.

AAA endangers the lives of nearly 10% of the elderly population and has a high mortality rate once ruptured [22]. For this reason, we have devoted resources to researching the related mechanisms of AAA over the past 3 years [23,24]. However, many factors affect the development of AAA. In 2022, the researchers found the presence of antibodies against *P. gingivalis*, along with elevated levels of LPS and high C-reactive protein (CRP) concentrations in all AAA patients. These findings suggest a systemic inflammatory and immune response associated with periodontal pathogens in individuals with AAA [25]. Our group examined the role of the GroEL protein from *P. gingivalis* in AAA formation in 2024. We discovered that GroEL promotes the secretion of MMP-2 from vascular smooth muscle cells through a process involving SUMOylation [26]. This mechanism enhances MMP-2 stability and activity, contributing to vascular remodeling and potentially accelerating AAA progression.

In AAA, macrophages can adopt a pro-inflammatory M1 phenotype or an anti-inflammatory M2 phenotype. The balance between these phenotypes influences disease progression. M1 macrophages contribute to inflammation and tissue degradation, promoting aneurysm formation, while M2 macrophages aid in tissue repair and resolution of inflammation. Studies have shown that modulating macrophage polarization can impact AAA development. For instance, elastin-derived peptides have been found to promote AAA formation by shifting macrophage polarization towards the M1 phenotype [27]. Similarly, in periodontitis, macrophage polarization is crucial in determining disease outcomes. The dynamic balance between these phenotypes affects the progression and resolution of periodontal disease. Research indicates that promoting M2 polarization can aid in resolving inflammation and supporting tissue regeneration in periodontal lesions [28]. Understanding the mechanisms that regulate macrophage polarization in these diseases may offer potential therapeutic targets for managing both AAA and periodontitis. Furthermore, platelets are a distinct population of blood-borne cellular components that contribute critically to vascular homeostasis. They are actively involved in processes such as hemostatic response to vascular injury, modulation of angiogenesis, and innate immune defense. Notably, extensive research has established a significant link between *P. gingivalis* and platelet function, underscoring its potential role in the pathogenesis of cardiovascular diseases [29].

Previously, we have found that the GroEL protein of *P. gingivalis* may induce systemic inflammation and exacerbate AAA formation by promoting the activation of vascular smooth muscle cells [26]. Since it has previously been established that monocytes/macrophages play important roles in the occurrence of AAA, in the present study, we also aimed to determine whether monocytes/macrophages are involved in GroEL-mediated *P. gingivalis*-related AAA formation and to elucidate the underlying mechanisms. To investigate this hypothesis, we employed a CaCl_2_-induced AAA mouse model combined with intravenous administration of recombinant GroEL to evaluate its in vivo effects. We further analyzed aortic tissue sections using immunohistochemistry and VVG staining to assess vascular remodeling and inflammatory infiltration. In parallel, immunofluorescence was used to examine macrophage phenotypes in aortic lesions. In vitro, THP-1 monocytic cells were stimulated with GroEL to evaluate changes in macrophage polarization, cytokine expression, and associated transcriptional regulators. This integrative approach allowed us to explore the immunopathological role of GroEL in AAA development.

## 2. Results

### 2.1. GroEL Accelerates AAA Formation in CaCl_2_ Immersion-Induced Mice

**Figure 1:**
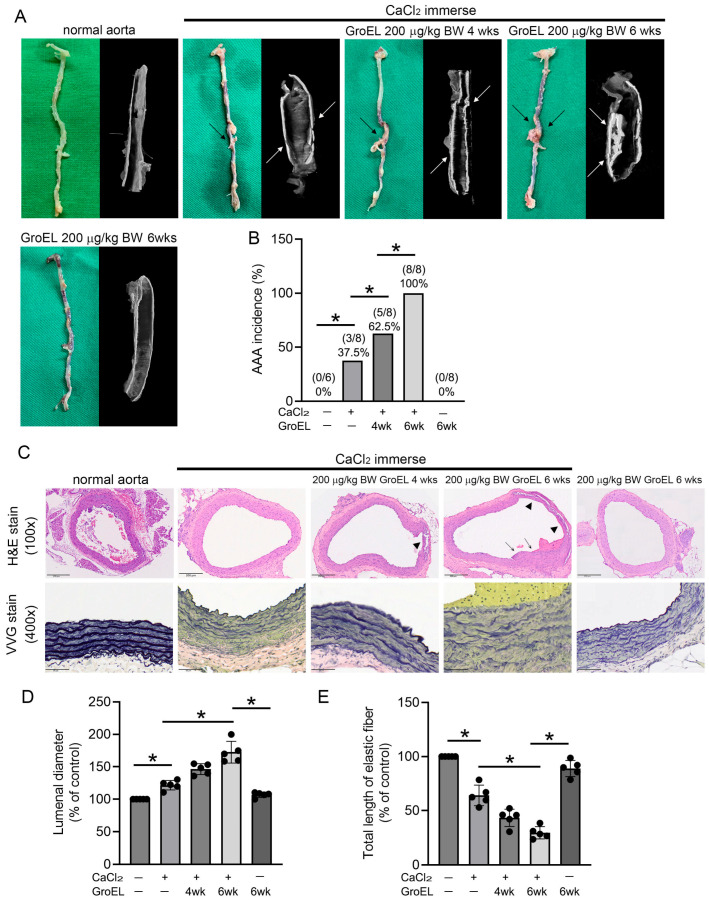
GroEL accelerates AAA formation in CaCl_2_ immersion-induced mice. (**A**) After the experiment, the total aortas of the rats were removed, and images were captured using a digital camera (left panel/green background). The black arrows indicate aneurysm lesions. The dissected abdominal aortas were then analyzed using micro-CT. Representative images of the tissues from each group are presented in the right panel (black background). The white arrows indicate vascular calcification and aneurysm lesions. (**B**) Graph presenting the AAA incidence of each group. (**C**) The upper column shows the abdominal aortas from rats stained with H&E. The images are 100× magnified. The arrowheads indicate aneurysm lesions. The lower column presents the integrity of the elastic fibers of abdominal aorta cross-sections observed using VVG staining. The images are 400× magnified. (**D**,**E**) Graphs displaying the quantification of the luminal diameter (**D**) and total length of elastic fibers (**E**). All the results in the graphs are expressed as the mean ± SD. * Statistical significance was set at *p* < 0.05.

We conducted a CaCl_2_ immersion-induced AAA animal experiment to ascertain the virulence of recombinant *P. gingivalis* GroEL in exacerbating AAA formation in vivo. To measure the possibility that the observed CaCl_2_ immersion-induced AAA formation was caused by GroEL, only the GroEL-treated control group was included in the experiments to ascertain the following results. Figure 1A shows photographs of the aortic appearance (green background) and vertical sections of the aortas (black ground) obtained using micro-CT in the experimental groups (Figure 1A). The normal aorta has an intact, smooth appearance and proper contrast. In contrast, immersion in CaCl_2_ induced slight AAA formation (black arrow) and increased the developing contrast in the vessel wall (white arrow). Notably, injection of GroEL (200 μg/kg BW) for 6 weeks resulted in more significant AAA formation and serious calcification in CaCl_2_ immersion-induced rats, although this phenomenon was not distinguished in the 4-week injection group. However, injection of GroEL alone (200 μg/kg BW) for 6 weeks did not induce AAA in rats. Figure 1B shows that the rate of AAA formation in rats after immersion in CaCl_2_ was 37.5%. This incidence of AAA reached 62.5% and 100% in rats that received an injection of 200 μg/kg BW GroEL for 4 and 6 weeks, respectively. However, injection of GroEL alone did not induce AAA formation. AAA manifests with vasculopathy, characterized by alterations in the vascular structure and endogenous collagen rupture [30]. Therefore, H&E and VVG staining were used to analyze the vascular structure and collagen phenomena. Through these methods, we observed that the normal aorta retained an intact morphology (H&E staining), even and visible collagen, and consistent and coherent elastic fibers (VVG stain). Moreover, although H&E staining showed that CaCl_2_ immersion did not induce observable damage to the aorta, VVG staining showed that this made the elastic fibers thinner. Furthermore, application of 200 μg/kg BW GroEL for 4 weeks caused changes in the appearance of the aortic structure (black arrowhead) and partial fracture of the elastic fibers, while CaCl_2_ immersion plus GroEL treatment induced severe collagen damage, especially after 6 weeks of treatment (Figure 1C). Figure 1D illustrates the variance in the luminal diameter of the abdominal aorta among the experimental animal groups, as determined by H&E staining. The results showed that administration of CaCl_2_ plus GroEL significantly increased the luminal diameter when compared to the CaCl_2_-only immersion group, although there was no difference between the CaCl_2_ immersion, GroEL injection only, and normal control groups (Figure 1D). Figure 1E depicts the disparity in the total length of the elastic fibers observed by VVG staining. Notably, the total lengths of elastic fibers in the CaCl_2_ immersion, CaCl_2_ immersion plus GroEL for 4 weeks, and CaCl_2_ immersion plus GroEL for 6 weeks groups were 64.1 ± 8.5%, 43.2 ± 7.2%, and 29.6 ± 5.0%, respectively, of that in the normal control group. In fact, the duration of GroEL administration was inversely proportional to the total length of the elastic fibers. These results suggest that *P. gingivalis* GroEL exacerbates CaCl_2_ immersion-induced AAA in rats.

### 2.2. GroEL Induces Higher Cytokine Production in CaCl_2_ Immersion-Induced AAA Rats

Chronic vascular inflammation is the main cause of AAA, with inflammatory mediators such as cytokines and chemokines playing critical roles [31]. Therefore, CRP, TNF-α, IL-1β, IL-6, IL-2, and INF-γ levels in the plasma of the experimental animals were analyzed using ELISA. As shown in Table 1, we observed no difference in the body weights of the experimental rats in each group before entering the experiment; moreover, there was no difference between the groups before the rats were sacrificed at the end of the second experimental week. Before entering the study, the baseline CRP levels were 31.3 ± 7.2, 37.3 ± 7.8, and 32.8 ± 9.4 mg/dL in the control, CaCl_2_ immersion, and CaCl_2_ + GroEL groups, respectively. Notably, CaCl_2_ immersion induced the elevation of CRP after 6 weeks of the experiment (from 218.0 ± 10.9 to 344.8 ± 12.4 mg/dL). Moreover, the administration of GroEL resulted in a more dramatic increase in CRP in CaCl_2_ immersion-induced rats (288.8 ± 19.8 mg/dL). CaCl_2_ immersion increased their respective concentrations (TNF-α: 940.0 ± 178.6 pg/mL, IL-1β: 105.2 ± 9.7 pg/mL, IL-6:39.9 ± 10.7 pg/mL, and INF-γ: 24.8 ± 4.3 pg/mL) when compared to the control (TNF-α: 55.3 ± 7.7 pg/mL, IL-1β: 73.8 ± 6.9 pg/mL, IL-6:27.1 ± 1.5 pg/mL, and INF-γ: 3.7 ± 1.1 pg/mL). The effects of CaCl_2_ immersion and CaCl_2_ immersion plus GroEL both resulted in higher levels of cytokine/chemokines (TNF-α, IL-1β, IL-6, and INF-γ), but GroEL addition only resulted in exacerbation of these responses (TNF-α: 1731.0 ± 119.5 pg/mL, IL-1β: 331.8 ± 53.6 pg/mL, IL-6: 291.7 ± 76.1 pg/mL, and INF-γ: 46.0 ± 13.9 pg/mL). However, the performance of IL-2 was not improved by CaCl_2_ immersion unless GroEL treatment was added, with the control and CaCl_2_ immersion groups having concentrations of 3.7 ± 1.0 pg/mL and 3.7 ± 0.6 pg/mL, respectively, and the CaCl_2_ + GroEL group having a concentration of 9.7 ± 2.0 pg/mL. These data indicate that CaCl_2_ immersion increases the systemic inflammatory response in rats, and GroEL administration exacerbates the inflammatory response in rats receiving CaCl_2_ immersion. This increased the levels of inflammatory cytokines associated with AAA development.

### 2.3. GroEL Induces Protein Expression Associated with Inflammation and AAA Formation in THP-1 Cells

**Figure 2:**
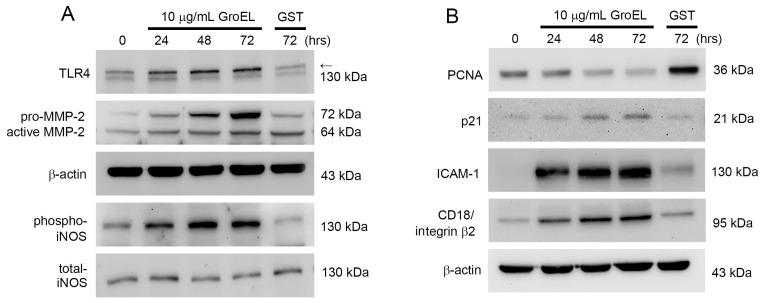
GroEL induces protein expression associated with inflammation and AAA formation in THP-1 cells. THP-1 cells were stimulated for 24–72 h with 10 μg/mL of GroEL. Treatment with 10 μg/mL of GST for 72 h served as a negative control. (**A**) Images showing the western blot analyses of TLR4, MMP-2, and phosphorylated iNOS proteins. (**B**) Images showing western blot analyses of PCNA, p21, ICAM-1, and CD18/integrin β2. The expression levels of total β-actin and total iNOS were used as loading controls.

Macrophage infiltration into the adventitia and media of the vessel wall plays a key role in AAA. Therefore, we used THP-1 cells to perform in vitro experiments to explore the effects of GroEL on this process. Notably, TLR4 may play a role in AAA formation through the recognition of *P. gingivalis* [32]. Additionally, many enzymes are involved in the process of aortic wall destruction, for which MMPs are the most important proteases [33], including MMP-2 [34]. Moreover, the activation of iNOS is involved in AAA development and progression [35]. As shown in Figure 2A, western blot analysis revealed that TLR4, MMP-2, and phosphorylated iNOS were slightly and spontaneously expressed at basal levels in control (non-GroEL-treated) THP-1 cells. Moreover, GroEL treatment for 24–72 h significantly upregulated the expression of TLR4, MMP-2, and phosphorylated iNOS in a time-dependent manner. Monocyte differentiation plays a critical role in AAA formation. In general, during monocyte/macrophage differentiation, PCNA expression decreases and p21 expression increases; therefore, we observed PCNA and p21 expression in THP-1 cells. The results showed that after 24–72 h of stimulation with 10 μg/mL GroEL in THP-1 cells, PCNA expression began to decrease and p21 expression began to increase (Figure 2B). Since adhesion molecules mediate the adhesion of monocytes to the endothelium, we predicted that ICAM-1 and CD18/integrin β2 on THP-1 cells may be involved in AAA formation. Our results showed that compared to a control group treated with 10 μg/mL GST protein, GroEL rapidly promoted the expression of ICAM-1 and CD18/integrin β2 and continued to support it for at least 72 h. This suggests that GroEL may induce inflammatory and differentiation responses in monocytes.

### 2.4. GroEL Induces M1 Macrophage Polarization and Inhibits IL-4/IL-13-Induced M2 Macrophage Polarization

**Figure 3:**
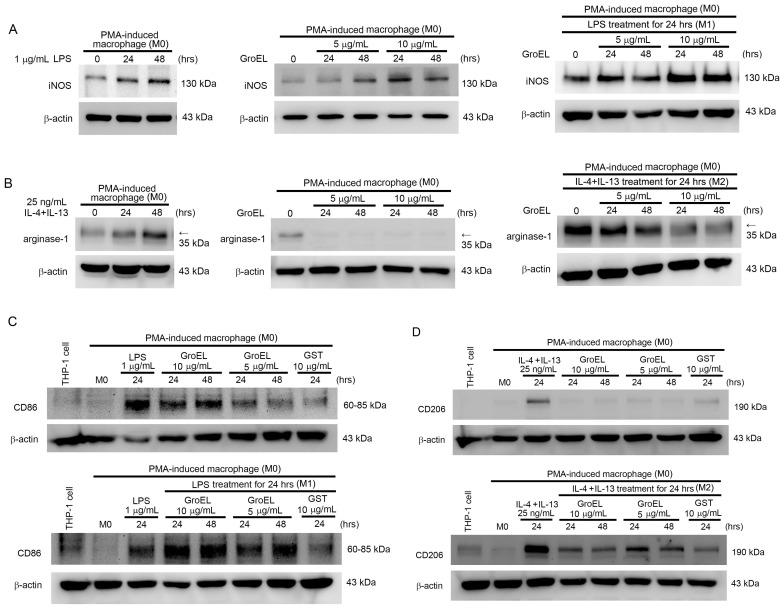
GroEL induces M1 macrophage polarization and inhibits M2 macrophage polarization. THP-1 cells were stimulated with 10 μM PMA for 24 h to differentiate into attached cells (M0 macrophages). (**A**) The left panel shows M0 macrophages stimulated with LPS. The middle panel shows M0 macrophages stimulated with GroEL. The right panel shows M0 macrophages stimulated with LPS first and GroEL afterwards. Total proteins were extracted, and western blot analysis of iNOS was performed. (**B**) The left panel shows M0 macrophages stimulated with 25 ng/mL IL-4 plus 25 ng/mL IL-13. The middle panel shows M0 macrophages stimulated with GroEL. The right panel shows M0 macrophages stimulated with IL-4 plus IL-13 first and GroEL afterwards. Total proteins were extracted, and western blot analysis of arginase-1 was performed. (**C**) The upper panel shows M0 macrophages stimulated with LPS or GroEL. The lower panel shows M0 macrophages stimulated with LPS with or without GroEL. Total proteins were extracted, and western blot analysis of CD86 was performed. (**D**) The upper panel shows M0 macrophages stimulated with 25 ng/mL IL-4 and 25 ng/mL IL-13 or GroEL; the lower panel shows M0 macrophages stimulated with IL-4 and IL-13 with or without GroEL. Total proteins were extracted, and western blot analysis of CD206 was performed. Treatment with 10 μg/mL GST was used to exclude the effect of the tagged protein on the recombinant GroEL protein. The expression of total β-actin was used as a loading control.

During AAA formation, monocytes differentiate into macrophages, infiltrate the aortic wall, and initiate inflammatory responses. Therefore, we analyzed the effect of GroEL on THP-1 cell differentiation using flow cytometry. We used the established phenomena of PMA-induced differentiation as a control. Table 2 presents the results of this experiment. Stimulation with 10 μM PMA for 48 h increased the proportion of THP-1 cells in the G0/G1 phase from 21.6 ± 5.5% to 56.9 ± 8.1%, while the S and G2/M phases decreased accordingly. Similarly, treatment with 5 or 10 μg/mL GroEL also significantly increased G0/G1 phase accumulation and reduced the proportions of cells in S and G2/M phases, suggesting that GroEL promotes monocyte differentiation in a manner comparable to PMA. A previous report demonstrated that LPS induces M1 macrophage polarization and expression of the inflammation-associated protein iNOS, whereas IL-4/IL-13 induces M2 macrophage polarization and produces the anti-inflammatory protein arginase-1 [36]. Figure 3A,B show that LPS may increase iNOS expression in M0 macrophages (Figure 3A, left), whereas treatment with IL-4 + IL-13 only induced arginase-1 expression in M0 macrophages (Figure 3B, left). Moreover, stimulating M0 macrophages with 5 and 10 μg/mL of GroEL, respectively, effectively induced the expression of iNOS (Figure 3A, middle). Furthermore, GroEL induced LPS-induced M1 macrophages to express more iNOS (Figure 3A, right). As shown in Figure 3B, M0 macrophages expressed arginase-1 at the basal level, while GroEL stimulation completely inhibited arginase-1 expression (Figure 3B, middle) and reduced IL-4/IL-13-induced M2 macrophage expression of arginase-1 (Figure 3B, right). However, other than CD86 and CD206, the markers currently used to detect M1 and M2 macrophages are still controversial. Therefore, we used these two markers to investigate macrophage polarization. As shown in Figure 3, LPS induced an increase in CD86 in M0 macrophages (M1 macrophage polarization), whereas GroEL promoted the polarization of M1 macrophages. In addition, the amount of CD86 expression was positively correlated with the concentration- and stimulation-time-dependent manner of GroEL (Figure 3C, upper). Moreover, when LPS-induced M1 macrophages were stimulated with GroEL, the expression of CD86 increased and reached a plateau (Figure 3C, lower). We also analyzed the effect of GroEL on M2 macrophage polarization. Figure 3D shows that IL-4/IL-13 can induce M0 macrophages, showing an increase in CD206 (M2 macrophage polarization); however, GroEL did not promote M2 macrophage polarization (Figure 3D, upper). When M2 macrophages induced by IL-4/IL-13 were stimulated with GroEL, the expression of CD206 was inhibited (Figure 3D, lower panel). Based on these results, we conclude that GroEL has the ability to both promote the polarization of M1 macrophages and enhance the polarization of M1 macrophages induced by LPS. We found that GroEL also promotes the expression of the inflammatory-response-related protein iNOS in M1 macrophages. In contrast, GroEL inhibited the polarization of M2 macrophages promoted by IL-4/IL-13 and reduced the expression of the anti-inflammatory protein arginase-1 in macrophages.

### 2.5. GroEL Induces M1 Macrophage Polarization via TM and IRF5 Expression

**Figure 4:**
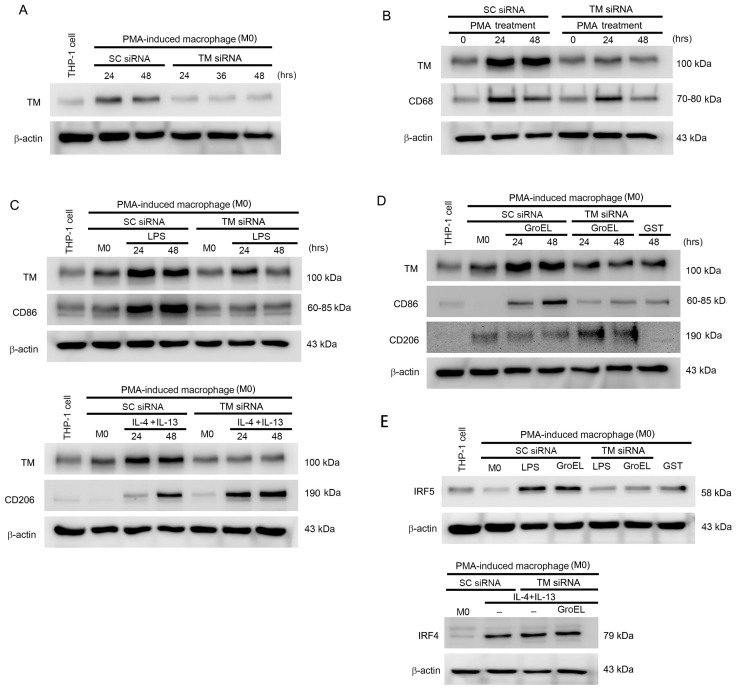
GroEL induces M1 macrophage polarization via TM and IRF5 expression. (**A**) THP-1 cells were stimulated with 10 μM PMA for 24 h to differentiate into attached cells (M0 macrophages), then cells were transfected with TM siRNA for 24–48 h, and the TM in total cell lysate was analyzed using western blot analysis. The scramble siRNA (SC siRNA) was used as a control. (**B**) THP-1 cells were transfected with or without TM siRNA, followed by 10 μM PMA treatment for 24–48 h. (**C**) THP-1 cells were stimulated with 10 μM PMA for 24 h to differentiate into M0 macrophages. The upper panel shows M0 macrophages transfected with or without TM siRNA, followed by 1 μg/mL LPS treatment for 24–48 h. The lower panel shows M0 macrophages transfected with or without TM siRNA, followed by 25 ng/mL IL-4 plus 25 ng/mL IL-13 treatment for 24–48 h. The total proteins were extracted, and western blot analyses of TM, CD68, CD86, and CD206 expressions were performed. (**D**) M0 macrophages were transfected with or without TM siRNA, followed by 10 μg/mL GroEL treatment for 24–48 h. (**E**) The upper panel shows M0 macrophages transfected with or without TM siRNA, followed by 1 μg/mL LPS or 10 μg/mL GroEL treatment for 48 h. The lower panel shows M0 macrophages transfected with or without TM siRNA, followed by 25 ng/mL IL-4 plus 25 ng/mL IL-13 combined with or without 10 μg/mL GroEL treatment. The total proteins were extracted, and western blot analyses of IRF5 or IRF4 expressions were performed. Treatment with 10 μg/mL GST for 48 h was used to exclude the effect of the tag protein on the recombinant GroEL protein. The expression of total β-actin was used as a loading control in this study.

Our previous study demonstrated that TM regulates monocyte differentiation in atherosclerotic arteries [37]. Therefore, we explored the effect of TM on M1 and M2 macrophage polarization. Figure 4A shows the efficiency of TM siRNA. As shown in Figure 4B, treatment with PMA for 48 h induced both TM and CD68 expression. Moreover, TM knockdown by TM small interfering ribonucleic acid (siRNA) effectively inhibited the increase in PMA-induced TM and CD68 expression when compared with the non-TM siRNA treatment groups at the same time point (Figure 4B). We also found that LPS induced M0 macrophages, showing an increase in CD86 at 24 and 48 h after stimulation (inducing M1 macrophage polarization). TM production was also increased by LPS stimulation, although it dropped back to basal levels at 48 h. When the TM of M0 macrophages was knocked down using siRNA, LPS did not increase the expression of CD86 in the cells (Figure 4C, upper). IL-4/IL-13 could also induce M0 macrophages, with an increase in CD206 at 24 and 48 h after stimulation (inducing M2 macrophage polarization). TM expression was also increased by IL-4/IL-13 stimulation. Notably, when the TM of M0 macrophages was knocked down, the expression level of CD206 remained high in M0 macrophages due to IL-4/IL-13 stimulation (Figure 4C, lower). These results show that both LPS and IL-4/IL-13 increase TM expression in macrophages. However, TM only participates in the process of M1 macrophage polarization. Knockdown of TM did not affect M2 macrophage polarization, which occurs in M0 macrophages under IL-4/IL-13 stimulation. We also analyzed whether TM is involved in GroEL-induced macrophage M1 polarization. Figure 4D shows that M0 macrophages stimulated by GroEL increased the expression of TM. The cells also increased the expression of CD86, but not CD206. After TM was knocked down, the increase in TM expression induced by GroEL was inhibited in M0 macrophages. Notably, the expression of CD86 only increased slightly, while that of CD206 increased significantly compared to cells without TM knockdown. It is well known that IRF5 and IRF4 are involved in the signaling pathways of macrophage M1 and M2 polarization, respectively [36]. As shown in Figure 4E, both LPS and GroEL increased IRF5 expression in M0 macrophages, whereas TM knockdown inhibited this increase in IRF5 activation in LPS- or GroEL-stimulated macrophages (Figure 4E, upper). Notably, M0 macrophages expressed low levels of IRF4. When these macrophages were stimulated by IL-4/IL-13, the expression of IRF4 increased regardless of whether or not TM was knocked down. Interestingly, the activation of IRF4 in macrophages was not affected by GroEL when TM was knocked down (Figure 4E, lower). These results suggest that TM and IRF5 play important roles in the differentiation of macrophages into M1 macrophages. In addition, they show that GroEL promotes macrophage M1 polarization through TM and IRF5 and inhibits macrophage M2 polarization promoted by IL-4/IL-13. In contrast, knockdown of TM may allow M0 macrophages to differentiate into M2 macrophages, reduce the influence of GroEL, and inhibit M1 macrophage polarization.

### 2.6. GroEL Accelerates AAA Formation in Ang II-Induced Mice Through Induction of M1 Polarization of Macrophages

**Figure 5:**
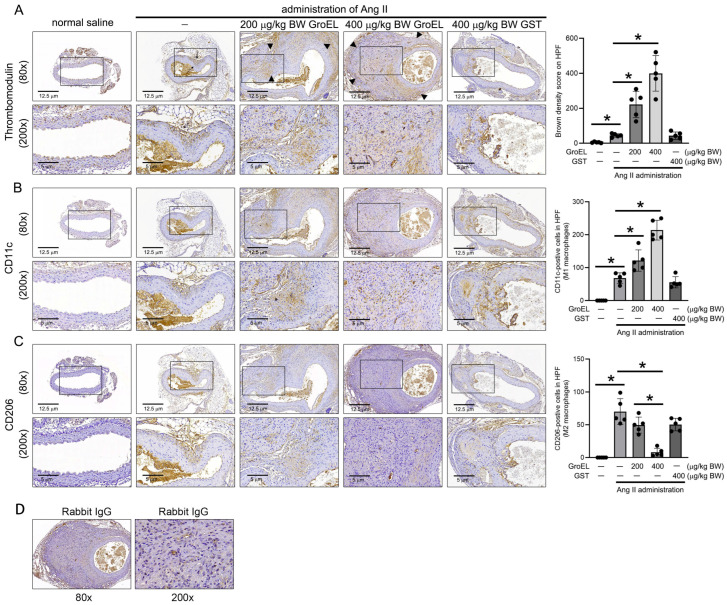
GroEL aggravates the occurrence of AAA, promotes infiltration of M1 macrophages, and inhibits polarization of M2 macrophages in the vessel walls in Ang II-induced mice. Images show aortic sections stained using (**A**) rabbit anti-TM Ab, (**B**) rabbit anti-CD11c Ab, (**C**) rabbit anti-CD206 Ab, and (**D**) isotype control rabbit IgG. In the upper panel, black arrows and arrow heads indicate aneurysm lesions in 80× magnification. The lower panel presents the boxes in the upper panels magnified at 200×. Bar graphs display the TM-expressed cells, CD11c-positive cells, and CD206-positive cells infiltrating the aneurysm lesion, counted under a high-power field (200×). All results in the graphs are expressed as the mean ± SD. * Statistical significance was set at *p* < 0.05.

To confirm the role of GroEL in exacerbating AAA formation in vivo through the induction of M1 polarization of macrophages, we observed macrophage differentiation in an Ang II-induced aortic remodeling animal experiment. We also included a GST-treated control group in these experiments to exclude the possibility that the observed effects were caused by the GST tags. Figure 5A shows the expression of TM in the abdominal aorta of experimental animals in each group. In the 80× magnification images at the top, it is clearly shown that the administration of Ang II induced mild arterial structural remodeling (black arrows) when compared with rats not treated with Ang II. Moreover, GroEL administration increased the severity of Ang II-induced AAA formation (arrowheads) in a dose-dependent manner, whereas GST administration did not increase the severity of Ang II-induced AAA. The lower panel of Figure 5A shows the TM expression in the blood vessels at 200× magnification, enlarging the boxed areas from the upper images. These images show that after Ang II administration, animals exhibited a slight increase in TM expression at the site of the arterial aneurysm (brown signal), and the administration of GroEL further increased TM expression in the AAA. The bar graph on the right shows that GroEL treatment increased TM expression in a dose-dependent manner. Figure 5B and Figure 5C show the infiltration of M1 and M2 macrophages, as indicated by CD11c and CD206, respectively, at the aneurysm lesion sites (brown signals). The results show that Ang II and GroEL influenced the infiltration of M1 and M2 macrophages at the aneurysm lesion site. In the high-power field (200×), we counted the infiltration of M1 and M2 macrophages in the aneurysm lesion and displayed these in the bar graph on the right side of the image. These results show that Ang II promoted the infiltration of M1 macrophages at the lesion site, and GroEL exacerbated M1 macrophage infiltration (Figure 5B). Similarly, Ang II promoted the infiltration of M2 macrophages at the lesion site, whereas GroEL suppressed M2 macrophage infiltration in Ang II-treated mice (Figure 5C). Notably, GST treatment did not alter the effect of Ang II on macrophage infiltration. The isotype control rabbit IgG was used to identify the specificity of antibodies (Figure 5D). Based on these results, it can be concluded that after four weeks of Ang II administration, visible AAAs were induced in mice, along with the promotion of M1 and M2 macrophage infiltration and increased TM expression at the lesion site. Additionally, GroEL administration exacerbated the formation of AAAs and infiltration of M1 macrophages while suppressing M2 macrophage infiltration in Ang II-induced mice. Thus, GroEL aggravates Ang II-induced vascular inflammation and inhibits the anti-inflammatory capacity of macrophages.

## 3. Discussion

AAAs are characterized by chronic vascular inflammation, smooth muscle cell apoptosis, neovascularization [38], and extracellular matrix degradation [33], primarily involving elastin and collagen. In particular, macrophages infiltrate the aortic wall [39] and release pro-inflammatory cytokines (e.g., TNF-α, IL-6, IFN-γ) and proteolytic enzymes such as MMPs [40], which contribute to medial degeneration. Elevated MMP activity, especially MMP-2 and MMP-9, is positively correlated with aneurysm diameter and severity, highlighting the importance of protease-mediated vascular remodeling. Collagen and elastin provide structural integrity to the aorta. In AAA patients, altered collagen turnover—particularly decreased pro-collagen type I and increased collagen type III—and the irreversible loss of elastin contribute to the weakening of the aortic wall [41]. These changes are exacerbated by inflammatory stimuli, many of which originate from infiltrating macrophages and activated VSMCs [42,43]. Our results confirm that GroEL aggravates elastin fragmentation and collagen disorganization, consistent with enhanced aneurysm formation. Macrophage polarization plays a critical role in AAA pathogenesis. M1 macrophages promote tissue destruction through the secretion of inflammatory mediators, whereas M2 macrophages facilitate repair. Our findings demonstrate that GroEL promotes M1 polarization while suppressing M2 polarization, suggesting a mechanism by which *P. gingivalis* contributes to AAA progression via immune modulation. Previous studies have shown that targeting macrophage function through genetic modulation can reduce inflammatory signaling and MMP expression in AAA models [44,45,46]. Our study further supports this concept by identifying GroEL as a microbial factor that skews macrophage responses toward the M1 phenotype. Thus, regulating macrophage polarization may be a promising therapeutic strategy to mitigate AAA development in individuals exposed to *P. gingivalis* or similar chronic infections [47].

TM was first identified in endothelial cells, where it binds to thrombin and activates protein C; in turn, this leads to the degradation of factors Va and VIIIa, achieving anticoagulation [48]. Elevated levels of TM have been detected in intact endothelial cells. However, when the endothelium is damaged, TM and thrombin on endothelial cells bind in large quantities and jointly regulate downstream coagulation. Thus, TM plays an extremely important role in inhibiting the excessive coagulation response after blood vessel damage. The combination of TM and thrombin can also activate thrombin-activatable fibrinolysis inhibitor, which regulates the degradation of C5a and C3a to achieve anti-inflammatory effects [49]. Recent studies have shown that TM has many functions that can be directly achieved through its lectin- and EGF-like domains. In addition to endothelial cells, keratinocytes [50], megakaryocytes, platelets [51], monocytes [52], neutrophils, smooth muscle cells [53], and cardiomyocytes [54] also have the ability to express TM. TM is also expressed in tumor cells and is related to tumor cell growth and metastasis [55,56]. Thus, the biological functions of TM are not limited to anticoagulation therapy. It has many diverse roles, including the regulation of the activation of coagulation factors [57], inhibition of the expression of cell adhesion molecules [58], and anti-inflammatory responses [59]. Abnormal TM performance is associated with the occurrence of many diseases, especially cardiovascular diseases [60]. In our previous research, we found that TM on the surface of monocytes was closely related to inflammation after cardiac surgery. In fact, patients undergoing coronary artery bypass graft surgery, who had higher levels of plasma TNF-α concentration, had a lower expression of TM on the surface of monocytes. This phenomenon leads to poor early outcomes after surgery [61].

We also found that the expression level of TM is related to the migration and differentiation of monocytes. When TM is decreased in monocytes, their migration ability increases, enhancing the inflammatory response and affecting the patient’s early outcomes [37]. TM also interacts with the cytoskeleton and inhibits activation of the MAPK/ERK and JNK/SAPK signaling pathways, thereby regulating the migration of monocytes [62]. Additionally, TM is related to the differentiation of monocytes [37] through the activation of the downstream MAPK/ERK pathway by combining with intracellular PKCδ, thereby activating p21 and inhibiting PCNA to promote cell differentiation (as shown in the upper-right picture [37]). In the present study, we demonstrated that GroEL promotes macrophage M1 polarization through TM and inhibits macrophage M2 polarization promoted by IL-4/IL-13. Moreover, we found that knockdown of TM may allow M0 macrophages to differentiate into M2 macrophages and can also reduce the influence of GroEL and inhibit M1 macrophage polarization. To our knowledge, this phenomenon has not been reported previously.

This study employed two distinct animal models—the Ang II-induced AAA model in mice and the CaCl_2_-immersion AAA model in rats—to comprehensively investigate the role of *P. gingivalis* GroEL in AAA formation through macrophage polarization. These models differ significantly in principle and application, allowing for a broader validation of GroEL’s role by addressing both systemic inflammatory mechanisms (Ang II model) and localized vascular injury (CaCl_2_ model). The Ang II-induced AAA model mimics AAA development under systemic conditions, including hypertension, oxidative stress, and lipid metabolism disorders. This model used genetically modified low-density lipoprotein receptor-deficient (LDLR^−/−^) mice, which are predisposed to lipid metabolism dysfunction. These mice were fed a high-fat diet and subjected to continuous Ang II infusion via mini-osmotic pumps, which promotes vascular inflammation, leukocyte infiltration, oxidative stress, and aneurysm formation, resembling human AAA associated with hypertension and atherosclerosis. This setup allowed the study to explore how *P. gingivalis* GroEL exacerbates AAA formation through M1 macrophage polarization, inflammatory cytokine signaling, and immune responses in a systemically inflamed environment. In contrast, the CaCl_2_-immersion AAA model involves direct chemical injury to the abdominal aorta by immersing the vessel in a CaCl_2_ solution, inducing localized inflammation, elastin degradation, smooth muscle apoptosis, and aneurysm formation. This model is particularly useful for studying morphological and structural changes, local inflammatory responses, and extracellular matrix remodeling. It focuses on localized tissue damage rather than systemic influences, providing insight into how *P. gingivalis* GroEL aggravates local inflammatory responses, promotes macrophage polarization, and accelerates extracellular matrix degradation. The activation of MMPs and the recruitment of inflammatory cells further contribute to aneurysm expansion in this model. By integrating these two experimental approaches, the study ensures mechanistic clarity, as the Ang II model highlights immune responses and cytokine signaling, while the CaCl_2_ model emphasizes vascular remodeling and extracellular matrix degradation. The findings provide robust evidence that *P. gingivalis* GroEL contributes to AAA formation via both systemic and local inflammatory pathways, offering valuable insights into potential therapeutic strategies for mitigating AAA progression.

## 4. Materials and Methods

### 4.1. Animal Study

#### 4.1.1. Induction of AAA in Rats Through CaCl_2_ Immersion

The induction of AAA in Sprague–Dawley (SD) rats through immersion in CaCl_2_ mirrors several pathological features observed in human AAA, including notable aortic calcification, vascular inflammation, oxidative stress, MMP production, elastin degradation, and vascular cell apoptosis. Previous investigations utilizing this model have elucidated the mechanisms underlying AAA development in rats, which may be relevant to human AAA [63]. Therefore, we implemented this CaCl_2_ immersion-induced AAA model in SD rats in the present study. Male SD rats, aged 10–12 weeks and weighing 300–350 g, were used in this study. We performed anesthesia procedures and exposure of the abdominal aorta according to previously described methods [64]. The abdominal aorta was then immersed in 0.5 M CaCl_2_ for 15 min, positioned between the renal artery and the iliac arteries. Subsequently, the organs were repositioned, and the incision was sutured. In the present study, 25 rats were randomly divided into five groups as follows: Group 1 (naïve control) rats did not receive surgical procedures and treatment; Group 2 (CaCl_2_) rats received CaCl_2_ immersion and were sacrificed at the end of week 6; Group 3 (CaCl_2_ + GroEL 200 μg/kg BW) rats received CaCl_2_ immersion, were administered an intraperitoneal injection of 200 μg/kg BW GroEL after surgery and thrice weekly throughout the experiment, and were sacrificed at the end of week 4; Group 4 (CaCl_2_ + GroEL 200 μg/kg BW) rats received CaCl_2_ immersion, were administered an intraperitoneal injection of 200 μg/kg BW GroEL after surgery and thrice weekly throughout the experiment, and were sacrificed at the end of week 6; and Group 5 (CaCl_2_ + glutathione S-transferase [GST] 200 μg/kg BW) rats received CaCl_2_ immersion, were administered an intraperitoneal injection of 200 μg/kg BW GST after surgery and thrice weekly throughout the experiment, and were sacrificed at the end of week 6. After the rats were sacrificed, the total aortas were removed and analyzed using a morphological assay.

#### 4.1.2. Induction of AAA in Mice Through Administration of Angiotensin II

Male B6.129S7-Ldlr^tm1 Her^/J mice (Stock No: 002077; JAX Labs, Farmington, CT, USA) were obtained from the Taiwan National Laboratory Animal Center (NLAC). This study utilized the mouse model of AAA developed by Poulsen et al. [65]. Eight-week-old male mice weighing 25–30 g were fed a high-fat diet (AIN-76A; St. Louis, MO, USA), starting on the first day of the experiment and continuing for 4 weeks (28 days). Subsequently, mini-osmotic pumps (Alzet Osmotic Pump 2004; Cupertino, CA, USA) were implanted. After anesthetizing the animals, their back hair was shaved, the skin was sterilized, and a 5-mm incision was made using sterile surgical scissors. Long forceps were used to create a non-adhesive space approximately 30 mm deep beneath the skin to implant mini-osmotic pumps containing either angiotensin II (Ang II; 1000 ng/kg/min) or normal saline. The wound was then sutured using 6-O silk. All surgical procedures were performed aseptically. The animals were sacrificed after being maintained on a high-fat diet for an additional 28 days after surgery. Twenty-five mice were randomly assigned to one of the five groups. Group 1 (saline control) rats received mini-osmotic pumps containing normal saline solution; Group 2 (Ang II) rats were administered mini-osmotic pumps containing Ang II; Group 3 (Ang II + GroEL 200 µg/kg BW) rats received mini-osmotic pumps with Ang II and intraperitoneal injections of 200 µg/kg BW GroEL after surgery, administered three times a week throughout the experiment; Group 4 (Ang II + GroEL 400 µg/kg BW) rats received mini-osmotic pumps with Ang II and intraperitoneal injections of 400 µg/kg BW GroEL under the same schedule; and Group 5 (Ang II + GST 400 µg/kg BW) rats received mini-osmotic pumps with Ang II and intraperitoneal injections of 400 µg/kg BW GST following the same regimen. At the end of the 4-week experiment, the mice were sacrificed and their aortas were harvested for subsequent analysis.

#### 4.1.3. Morphological Analysis Using Hematoxylin and Eosin Staining and Micro-Computed Tomography

After the animals were euthanized, the total aortas (thoracic and abdominal) were dissected for photography. The abdominal aortas were collected and fixed by immersion in 4% paraformaldehyde (Sigma-Aldrich, St. Louis, MO, USA). Subsequently, paraffin (Sigma-Aldrich, St. Louis, MO, USA) embedding was performed, followed by micro-computed tomography (CT) using a Bruker SkyScan 1276 (Bruker Corp., Billerica, MA, USA). The tissues were then sectioned into 5 μm thick sections. Morphological changes in the vessels were examined using hematoxylin and eosin (H&E) staining.

#### 4.1.4. Immunohistochemistry

Immunohistochemical (IHC) staining was performed on 5 μm thick aortic sections using rabbit antibodies against CD11c (GTX32507; GeneTex, Taipei, Taiwan), CD206 (ab-64693; Abcam Inc., Cambridge, MA, USA), and thrombomodulin (TM; PA5-120883; Invitrogen Inc., Los Angeles, CA, USA). The isotype control rabbit IgG was used to identify the quality of antibodies. Stained tissue slides were observed and scanned using a light microscope (BX50; Olympus, Tokyo, Japan) equipped with a MicroVisioneer Scanner (Cell-Bio Biotech, Taipei, Taiwan), and the numbers of CD11c-positive and CD206-positive cells were calculated.

#### 4.1.5. Enzyme-Linked Immunosorbent Assay

Plasma inflammatory cytokines were analyzed using an enzyme-linked immunosorbent assay (ELISA). The ELISA kit used to analyze C-reactive protein (CRP) was purchased from Abcam Inc. (Cambridge, MA, USA), while the kits used to analyze tumor necrosis factor (TNF)-α, IL-1β, IL-2, and interferon (IFN)-γ were purchased from Invitrogen Inc. (Los Angeles, CA, USA).

#### 4.1.6. Verhoeff–Van Gieson Staining and IHC

Verhoeff–Van Gieson (VVG) and Sirius Red staining were used to stain the elastic fibers and collagen in the vessels. These stained tissue slides were observed and scanned using a light microscope (BX50; Olympus, Tokyo, Japan) equipped with a MicroVisioneer Scanner (Cell-Bio Biotech, Taipei, Taiwan).

### 4.2. In Vitro Study

#### 4.2.1. Cultivation of THP-1 Cells

THP-1 cells, a human pro-myelomonocytic cell line, were acquired from the American Type Culture Collection (ATCC, Manassas, VA, USA) and cultured in RPMI 1640 medium (Thermo Fisher Scientific Inc., San Francisco, CA, USA) supplemented with 2 mM L-glutamate, 4.5 g/L glucose, 10 mmol/L HEPES, 1.0 mmol/L sodium pyruvate, 10% fetal bovine serum, and 1% antibiotic-antimycotic mixture. Cell density was maintained within the range of 5 × 10^4^ to 8 × 10^5^ viable cells/mL, and the medium was replenished every 48–72 h. To induce differentiation into a macrophage-like phenotype identified as an M0 macrophage, cells were treated with 10 μM phorbol 12-myristate 13-acetate (PMA) in complete medium for 24 h at 37 °C in 5% CO_2_.

#### 4.2.2. Production and Purification of Recombinant *P. gingivalis* GroEL Protein

The method for preparing recombinant *P. gingivalis* GroEL has been described in our prior publications [19,21]. Pierce High-Capacity Endotoxin Removal Spin Column (Thermo Fisher Scientific Inc., San Francisco, CA, USA) was used to clean the LPS contamination. Given the use of GST as a reference protein for recombinant protein preparation, recombinant GST was synthesized for use in the control group in the present experiment. Quantification of the concentration and purity of recombinant proteins was conducted using a Bio-Rad protein assay (Bio-Rad, Hercules, CA, USA) in conjunction with sodium dodecyl sulfate-polyacrylamide gel electrophoresis (SDS-PAGE), followed by Coomassie blue staining.

#### 4.2.3. Knockdown of TM Gene Expression by siRNA Transfection

To suppress TM gene expression, siRNA transfection was performed. A total of 1 × 10^6^ THP-1 cells were suspended in 2.5 mL of serum-free medium and exposed to 25 μM TM siRNA duplexes (Invitrogen, Catalog: THBD-HSS110719, 110721, 186320, Grand Island, NY, USA) for 6 h. The transfection was carried out using Lipofectamine^®^ RNAiMAX (Invitrogen, Carlsbad, CA, USA) in accordance with the manufacturer’s guidelines. Afterward, the cells were transferred to complete medium and incubated for 24 h. The knockdown efficiency of TM expression was then assessed via western blot analysis.

#### 4.2.4. Flow Cytometry for Analysis of Cell Differentiation

To perform flow cytometry, THP-1 cells were fixed with 70% alcohol and stained with propidium iodide (PI) dye (25 μg/mL PI, 40 μg/mL ribonuclease, and 0.3% Tween-20). Subsequently, to analyze the cell differentiation cycle, the DNA content in the cells was counted using a FACSCalibur^TM^ Flow Cytometer (BD Biosciences, Franklin Lakes, NJ, USA).

#### 4.2.5. Western Blot Analysis for Protein Production

Western blot analysis was used to examine protein expression. First, total protein extracts were separated using SDS-PAGE. After electrophoresis, the gel was transferred onto a polyvinylidene fluoride membrane. Subsequently, immunoblotting was conducted using rabbit anti-toll-like receptor 4 (TLR4) antibodies (Ab) (ab-13566; Abcam, Waltham, MA, USA), rabbit anti-CD206 Ab (ab-64693; Abcam, Waltham, MA, USA), rabbit anti-TM Ab (ab-109189; Abcam, Waltham, MA, USA), rabbit anti-IRF4 Ab (ab-228442; Abcam, Waltham, MA, USA), rabbit anti-IRF5 Ab (ab-21689; Abcam, Waltham, MA, USA), rabbit anti-MMP-2 Ab (sc-10736; Santa Cruz Biotechnology, Dallas, TX, USA), mouse anti-β-actin Ab (A-5441; Sigma-Aldrich, Burlington, MA, USA), rabbit anti-total-inducible nitric oxide synthase (iNOS) Ab (#20609; Cell Signaling Technology, Danvers, MA, USA), rabbit anti-phospho-iNOS Ab (#9571; Cell Signaling Technology, Danvers, MA, USA), rabbit anti-p21 Ab (#2947; Cell Signaling Technology, Danvers, MA, USA), rabbit anti-arginase Ab (#93668; Cell Signaling Technology, Danvers, MA, USA), rabbit anti-CD86 Ab (#91882; Cell Signaling Technology, Danvers, MA, USA), rabbit anti-CD68 Ab (#86985; Cell Signaling Technology, Danvers, MA, USA), rabbit anti-proliferating cell nuclear antigen (PCNA) Ab (GTX100539; GeneTex, Irvine, CA, USA), goat anti-ICAM-1 Ab (BBA17; R&D Systems, Minneapolis, MN, USA), rabbit anti-CD18/integrin β2 Ab (MBP1-88127; Novus Biologicals, Centennial, CO, USA), HRP-conjugated anti-rabbit immunoglobulin G (IgG), and HRP-conjugated anti-goat IgG, sequentially. To assess alterations in protein expression, membranes were visualized using Immobilon Western Chemiluminescent HRP Substrate (WBKLS Millipore; Merck KGaA, Darmstadt, Germany) and imaged using the BioSpectrum 500 Imaging System (WolfLabs, Pocklington, UK).

### 4.3. Statistical Analyses

Values are expressed as mean ± standard deviation (SD). One-way analysis of variance (ANOVA) and post hoc tests were used to conduct statistical analyses, followed by Tukey’s test. Statistical significance was set at *p* < 0.05.

## 5. Conclusions

We found that *P. gingivalis* GroEL exacerbated AAA formation. We also observed that TM contributes to this mechanism through GroEL-mediated M1 polarization of monocytic cells. Specifically, GroEL stimulation upregulated TM expression in THP-1 cells, which in turn promoted IRF5 activation and enhanced M1-associated protein expression, thereby facilitating monocyte differentiation. In contrast, TM knockdown did not affect M2 polarization. This study demonstrates a previously unrecognized role of TM in mediating inflammatory macrophage responses. Our findings reveal a novel mechanistic link between *P. gingivalis* GroEL and AAA progression through the modulation of monocyte/macrophage polarization. By identifying TM as a key intermediary in this process, this study offers new insights into how microbial factors contribute to vascular inflammation and suggests potential therapeutic targets for preventing or attenuating AAA development in patients with chronic infections.

## 6. Limitations

Despite providing new insights into the role of *P. gingivalis* GroEL in AAA pathogenesis, this study has several limitations. First, the CaCl_2_-induced AAA model used here primarily reflects localized inflammation and medial degradation, but it may not fully replicate the complex hemodynamic and genetic factors observed in human AAA. Second, although THP-1 cells provide a well-established in vitro system for studying macrophage polarization, they are a monocytic leukemia cell line and may not completely mirror the behavior of primary human monocytes/macrophages. Third, the mechanistic role of TM was inferred based on gene knockdown and in vitro assays, and further in vivo validation, such as through conditional TM knockout mice, will be necessary to confirm its functional relevance in AAA development. Additionally, although our findings demonstrate that GroEL significantly exacerbates AAA progression in the CaCl_2_-induced mouse model, it is important to note that GroEL alone was not sufficient to induce AAA formation in the absence of vascular injury. This suggests that GroEL requires a pre-existing pro-inflammatory or structurally compromised vascular environment to exert its pathogenic effects. The CaCl_2_ model simulates medial degradation and localized inflammation, conditions under which GroEL may further amplify immune responses and tissue damage. However, without such a baseline injury, GroEL does not appear to initiate aneurysmal remodeling on its own. This limitation highlights the importance of tissue context in microbial factor-induced vascular pathology and suggests that GroEL may act more as a disease enhancer than a primary initiator. Further studies using alternative models or long-term GroEL exposure could help clarify its role in aneurysm initiation. Lastly, this study focused mainly on macrophage-mediated inflammation; other immune cell types and systemic interactions were not addressed and warrant future investigation.

## Figures and Tables

**Table 1:** Comparison of cytokines in CaCl_2_-immersed abdominal aorta Rats.

	Control (Naïve)		CaCl_2_ Immerse		CaCl_2_ Immerse Plus 200 μg/kg BW GroEL	
	Baseline	6 Weeks	Baseline	6 Weeks	Baseline	6 Weeks
Body weight (g)	305.8 ± 9.8	340.3 ± 9.5	318.0 ± 10.9	344.8 ± 12.4	311.3 ± 8.0	334.8 ± 15.0
CRP (mg/dL)	31.3 ± 7.2	40.8 ± 9.1	37.3 ± 7.8	149.5 ± 29.3 ^ab^	32.8 ± 9.4	288.8 ± 19.8 ^abc^
TNF-α (pg/mL)	67.9 ± 14.3	55.3 ± 7.7	52.1 ± 24.9	940.0 ± 178.6 ^ab^	41.7 ± 22.2	1731.0 ± 119.5 ^abc^
IL-1β (pg/mL)	75.5 ± 5.8	73.8 ± 6.9	80.1 ± 6.7	105.2 ± 9.7 ^ab^	77.0 ± 7.3	331.8 ± 53.6 ^abc^
IL-6 (pg/mL)	28.8 ± 4.3	27.1 ± 1.5	27.1 ± 3.8	39.9 ± 10.7 ^ab^	29.7 ± 2.0	291.7 ± 76.1 ^abc^
IL-2 (pg/mL)	3.5 ± 0.8	3.7 ± 1.0	3.5 ± 0.7	3.7 ± 0.6	3.5 ± 0.8	9.7 ± 2.0 ^abc^
INF-γ (pg/mL)	3.5 ± 1.0	3.7 ± 1.1	3.2 ± 0.6	24.8 ± 4.3 ^ab^	3.2 ± 0.5	46.0 ± 13.9 ^abc^

CRP, C-reactive protein; TNF-α, tumor necrosis factor-alpha; IL-1β, interleukin-1 beta; IL-6, interleukin-6; IL-2, interleukin-2; INF-γ, Interferon gamma. Value is presented as mean ± SD. ^a^ *p* < 0.05 compared to baseline in the same group; ^b^ *p* < 0.05 compared to the control group at the same time point; ^c^ *p* < 0.05 compared to the CaCl_2_ immerse group at the same time point.

**Table 2:** The DNA content of THP-1–differentiated macrophages after GroEL administration for 48 h.

	Control	PMA	5 μg/mL GroEL	10 μg/mL GroEL
G_0_/G_1_ phase	21.6 ± 5.5%	56.9 ± 8.1% ^*p* < 0.05^	64.1 ± 1.9% ^*p* < 0.05^	68.4 ± 9.1% ^*p* < 0.05^
S phase	55.7 ± 7.2%	29.7 ± 6.4% ^*p* < 0.05^	20.4 ± 5.6% ^*p* < 0.05^	19.4 ± 2.3% ^*p* < 0.05^
G_2_/M phase	16.5 ± 6.1%	6.4 ± 3.1% ^*p* < 0.05^	6.6 ± 2.3% ^*p* < 0.05^	7.2 ± 3.1% ^*p* < 0.05^

PMA, phorbol myristate acetate. Values are presented as mean ± SD. *p* < 0.05 compared to the control group at the same phase.

## Data Availability

Data is contained within the article.

## Abbreviations

The following abbreviations are used in this manuscript:

AAA	abdominal aortic aneurysm
Ang II	angiotensin II
CRP	C-reactive protein
eNOS	endothelial nitric oxide synthase
GST	glutathione S-transferase
HSP60	heat shock protein 60
H&E	hematoxylin and eosin
iNOS	inducible nitric oxide synthase
ICAM	intercellular adhesion molecule
IRF5	interferon regulatory factor 5
IFN-γ	interferon gamma
IL	interleukin
LPS	lipopolysaccharide
MMP	metalloproteinase
PMA	phorbol myristate acetate
*P. gingivalis*	*Porphyromonas gingivalis*
PCNA	proliferating cell nuclear antigen
SMCs	smooth muscle cells
TM	thrombomodulin
TLR4	toll-like receptor 4
VVG	Verhoeff–Van Gieson
